# Unmasking factors affecting sleep pattern disturbances among patients undergoing hemodialysis: A cross-sectional study

**DOI:** 10.1371/journal.pone.0351651

**Published:** 2026-06-17

**Authors:** Hala Sanad, Khaldoon Al-Roomi, Zahra Abdlmjeed, Mariam Ebrahim, Omar Al Omari, Rasha Eweida, Mohamed Elsehrawy, Khulood Alasfoor, Yusuf Altahoo, Eshrak Hashem, Ahmed El-Monshed, Magda Bayoumi

**Affiliations:** 1 Department of Nursing, College of Health and Sport Sciences, University of Bahrain, Manama, Bahrain; 2 Department of Family and Community Medicine, College of Medicine and Health Sciences, Arabian Gulf University, Manama, Bahrain; 3 Hemodialysis Unit, A. Rahman Kanoo Center for Kidney Diseases, Government Hospitals - Ministry of Health, Muharraq, Bahrain; 4 Fundamentals and Administration Department, College of Nursing, Sultan Qaboos University, Seeb, Oman; 5 Department of Psychiatric and Mental Health Nursing, Faculty of Nursing, Alexandria University, Alexandria, Egypt; 6 Nursing Administration and Education Department, College of Nursing, Prince Sattam Bin Abdulaziz University, Al-Kharj, Saudi Arabia; 7 Nursing Administration Department, Faculty of Nursing, Port Said University, Port Fuad, Egypt; 8 Family Medicine Department, Shaikh Salman Health Center, Muharraq, Bahrain; 9 Family Medicine Department, Aster Medical Center, Capital, Bahrain; 10 Medical -Surgical Nursing Department, Faculty of Nursing, Alexandria University, Alexandria, Egypt; 11 North Private College of Nursing, Arar, Saudi Arabia; 12 Department of Psychiatric and Mental Health Nursing, Faculty of Nursing-Mansoura University, Mansoura, Egypt; 13 Department of Medical -Surgical Nursing & Critical Care, Faculty of Nursing, Bani-Suef University, Bani-Suef, Egypt; Japanese Red Cross Medical Center, JAPAN

## Abstract

**Background:**

The number of patients on hemodialysis is steadily increasing in Gulf Arab countries, including Bahrain. Such treatment modality has a negative impact on the quality of life, particularly sleep quality. This study aimed to assess the factors affecting the sleep pattern disturbances of hemodialysis patients in Bahrain.

**Materials and Methods:**

A cross-sectional design was employed. A convenience sample of 174 patients was recruited to the study from one of the main dialysis centers in Bahrain. Data was obtained via the Pittsburgh Sleep Quality Index questionnaire. Both univariate and multivariate analyses were used. A p value of less than 0.05 was considered statistically significant.

**Results:**

Most of the patients had poor sleep patterns (91%). It would appear that the main contributing factors are older age (p=0.009), self-rated health (p=0.001), anemia (p=0.002), excessive weight gain, and pain symptoms after dialysis session (p=0.001, p=0.001 respectively) and taking medications for sleep (p=0.016).

**Conclusion:**

There is an extremely high level of poor sleep quality among patients on hemodialysis. The contributing factors are multifactorial. There is an urgent need to include sleep assessment, mental health screening, and psychosocial support education into the routine nephrology care program.

## Introduction

Chronic kidney disease (CKD) is an important global public health medical problem, affecting around 10% of the population worldwide and leads to millions of deaths each year because of limited access to affordable treatment [1]. The rising prevalence of this condition increases the burden on healthcare systems. The prevalence and incidence of CKD have increased by 40% over the past three decades, which can be related to the growth and ageing of the global population [2]. In 2021, the global number of CKD cases reached an estimated 673.7 million, with 11.13 million new cases and 1.53 million deaths recorded [3–4]. Patients with CKD on hemodialysis suffer difficulties that go beyond the physiological aspects of their condition. The treatment itself can impose both physiological and psychological pressures, which clearly impact their sleep quality [5–6]. Furthermore,these individuals mostly experience physical discomfort, increased stress levels, and are required to make substantial lifestyle modifications to accommodate their treatment regimen [7]. Notably, individuals with kidney failure exhibit the highest financial and symptom burdens, along with the lowest scores in health-related quality of life. Therefore, addressing challenges encountered by hemodialysis patients is helpful in improving their well-being and ensuring better health outcomes [8].

The prevalence of sleep pattern disturbances is high in patients on hemodialysis. Studies show high rates of sleep problems among these patients, with prevalence ranging from 40% to 80% [9–10]. Sleep problems are strikingly common among patients undergoing regular hemodialysis treatment; sleep disturbance rates were reported as high as 63.73%, showing the impact of sleep-related issues in this clinical group [11]. Sleep problems affect patients undergoing dialysis in the Middle East. In Egypt, for example, research has found that 80% of patients are experiencing poor sleep.. Moreover, insomnia was found to be a major complaint in 66% of patients in that study. This shows that sleep disturbances are a major issue among patients on dialysis [12].

Sleep problems lead to a decrease in the quality of life for patients on hemodialysis and an increased risk of cardiovascular disease. Poor sleep quality in this population leads to elevated likelihood of both morbidity and death and suggests that addressing sleep issues could enhance survival rates [13]. Chronic sleep deprivation can also lead to a weak immune system, an increased susceptibility to cardiovascular complications, and impairments in cognitive function [14]. Moreover, sleep disturbances can facilitate the emergence or exacerbation of mental health disorders such as depression and anxiety, diminishing the overall well-being of these patients [15]. The impact of poor sleep extends to daily functioning, potentially hindering patients’ ability to care for their families and engage actively in social life, thereby markedly diminishing their overall quality of life [16].

Across the Middle East, there are huge differences in the number of people who receive treatment for kidney failure. The numbers range dramatically, from just 53 patients per million in some countries to over 400 per million in others. These variations show how unevenly kidney care is distributed throughout the region [17]. Recent data from Bahrain paints a concerning picture: over 16% of adults living with type 2 diabetes have developed moderate to severe chronic kidney disease (stages 3–5) [18]. In the presence of a high prevalence of diabetes in the Gulf Cooperation Council countries, this is particularly relevant [19]. A survey of renal replacement therapy in Bahrain revealed that out of 536 patients, 89.2% were undergoing hemodialysis, highlighting the significant reliance on this treatment modality in the country [20].

The interest in conducting research on factors affecting sleep pattern disturbances in hemodialysis patients within the Kingdom of Bahrain arises from the unique context of the country. 87.5% of the population in Bahrain is in the economically productive age group between 15 and 64 years [21]. Ageing may lead to an increase in patients with CKD and hemodialysis, which might reveal factors influencing sleep patterns that differ from other populations. Furthermore, environmental cultural factors, and protocols used for the management of hemodialysis patients in Bahrain that can determine sleeping habits and quality among hemodialysis patients, which might be different from other regions in which other studies have been conducted. It is important to investigate these local healthcare practices for developing relevant interventions.

A potential gap exists in the understanding of sleep disturbances within the Bahraini hemodialysis population. Even though data on the prevalence of CKD and hemodialysis in Bahrain is available [22], there is a lack of dedicated research investigating the specific factors that contribute to sleep pattern disturbances in this patient group. Although a study on nonsteroidal anti-inflammatory drug (NSAID) prescription among CKD patients in Bahrain demonstrates ongoing research in the broader area of kidney disease, it does not directly address the issue of sleep [23].

The potential implications of this study’s outcomes for informing clinical interventions and enhancing patient care within the Kingdom of Bahrain are significant. The identification of key factors influencing sleep disturbances will enable clinicians to develop more targeted and effective interventions. The study’s findings can also help in the development of culturally appropriate educational materials and programs for both patients and healthcare providers, not only in Bahrain but also in countries with a similar population and healthcare systems.

This study aimed to assess the factors affecting the sleep pattern disturbances of hemodialysis patients in Bahrain.

Research objectives:

1. Identify the level of sleep pattern disturbances among hemodialysis patients in Bahrain.
2. Explore the factors that affect the sleep pattern among hemodialysis patients in Bahrain.
3. Investigate the relationship between demographic factors and clinical characteristics of sleep disturbances in hemodialysis patients

## Materials and methods

### Study design and setting

A descriptive survey cross-sectional design was employed to assess the quality of sleep among hemodialysis patients in Bahrain. The study was conducted at the hemodialysis units of Abdulrahman Kanoo Dialysis Centre (AKDC) located in the Muharraq governorate in Bahrain (a major dialysis center in Bahrain). AKDC is a government medical center equipped with 48 beds that provides free services and treatment to all Bahraini citizens who require hemodialysis or peritoneal dialysis.

### Sampling and sample size

A convenience sample of 174 participants was enrolled in the study. The inclusion criteria were adults aged 18 or older, receiving at least three regular hemodialysis sessions, and able to understand the Arabic language. Patients with a history of cognitive impairment or a mental illness that may impact their ability to complete the questionnaires, comorbid obese patients, and the key principle was to exclude patients taking medications known to significantly affect sleep. Accordingly, sedatives/hypnotics, opioids, and sedating antidepressants were excluded. Analgesics that do not substantially alter sleep architecture—such as paracetamol and nonsteroidal anti‑inflammatory drugs (NSAIDs)—were generally permitted; however, their use was still evaluated in accordance with the study’s eligibility criteria. The sample size was calculated based on an estimate of a 95% confidence interval for the mean knowledge score with 5.0 width and 15.0 standard deviation using the single mean equation for sample size and accounting for a non-response rate of about 10%, which indicated a minimum sample size of around 170 patients [24].

### Data collection tools

#### Tool I: Socio-demographic and clinical structured tool.

The questionnaire in the Arabic language included two parts and consisted of twenty-eight questions. Part one, which covered sociodemographic data included age, gender, marital status, number of siblings, education level, income, and employment status. Part two is the clinical structure-based questionnaire. It was developed by researchers and included information that could potentially influence or affect the patients’ sleep, such as smoking status, the dialysis sessions per week, type of catheter, pain assessment, chronic disease, and psychiatric diseases.

#### Tool II: The Pittsburgh Sleep Quality Index (PSQI).

PSQI is widely used as a reliable tool to assess sleep quality and disturbances over a one-month time interval among patients on hemodialysis [25]. The tool included seven components: subjective sleep quality, sleep latency, sleep duration, habitual sleep efficiency, sleep disturbances, use of sleep medication, and daytime dysfunction. The tool sought information on the frequency of sleep disturbances according to the categories of: Not during the past month, less than once a week, once or twice a week, and three or more times a week. The tool included a total of 24 questions, divided into four open-ended questions and 20 closed-ended items that were rated with 0 representing (having no trouble at all), 1 (having trouble once), 2 (having trouble twice), and 3 (having trouble three times). The total score ranged from 0 to 21, a higher score indicates low sleep quality, whereas a low score indicates normal sleep quality. Patients who scored less than 5 were considered to have normal sleep, and patients who scored 5 or more were considered to have poor sleep. The tool has been translated into Arabic and has been used in a previous study of patients diagnosed with renal failure. The Arabic version showed adequate validity and reliability for assessing sleep quality [26].

### Procedure

After obtaining official approval from the research committee at government hospitals to carry out this study, data were collected from participants via an interviewer-administered questionnaire where responses were recorded using an online Google form during the scheduled hemodialysis session. Consent was obtained from the participants after explaining the aim of the study. Two researchers were responsible for collecting the data.

### Statistical analysis

SPSS software was used to conduct statistical analysis (Statistical Package for Social Science, Version 23). The demographic characteristics were described using frequencies and percentages statistics depending on the level of measurement. Mean and standard deviation were employed as descriptive statistics to define the scores of sleep quality among demographic parameters. The ANOVA test and independent t-test were used to describe the relationship between Sociodemographic characteristics and sleep quality. The Spearman correlation test was used to determine and measure the correlation between sleep quality and different study variables. Multiple linear regression analysis was used to determine predictors of sleep quality. P values a two-tailed test were considered statistically significant if they were less than 0.05.

### Ethical considerations

Ethical approval for this project was obtained from the Research Committee at the Government Hospitals (RCGH), Kingdom of Bahrain (No 55230522). The study followed the guidelines of the Helsinki Declaration. Verbal consent was obtained from the study participants after a comprehensive explanation of the study’s aim and objectives. This was witnessed by the nurse in charge of the hemodialysis unit and documented by a member of the research team. The RCGH approved the verbal consent approach and thought that it was more appropriate in this situation since obtaining a written consent from these patients would be difficult in view of the limited literacy and the cultural sensitivity in signing written documents. Further, the researchers explained to the participants that they had the right to refuse, participate or withdraw from the study at any time without jeopardizing their right to the treatment. Information obtained from patients was securely stored. In addition, the confidentiality of the data was assured and analyzed as a total sample and not as individuals. Thus, the data remained anonymous and used solely for research purposes.

## Results

Table 1 shows that around three-quarters of the studied patients were over 50 years (72.4%), with more than half of them being male (54%). The majority of patients were married and responsible for themselves and their families (79.3%). More than one-third of subjects (39.7%) have 4–6 children. Moreover, 83.3% of the participants do not work, and 67.2% of them could be the family breadwinner. The majority of the subjects have enough income (90.2%). 79.9% of the studied patients have Secondary education or less. 54.6% of the participants have daily home activities, and only 23% of patients reported that the location of their residence has an impact on disturbing their sleep. The mean weight in KG of the subjects was 76.51 ± 17.07. There is a statistically significant relationship between sleep quality with age and weight of patients (p = 0.009, 0.002), respectively.

**Table 1:** Socio-demographic Characteristics and their relation to sleep quality among study participants (n = 174).

Variable	N	%	Sleep quality	F	P value
**Age**					
Less than 30 years	7	4.0	10.43 ± 1.9		
30: < 40 years	12	6.9	9.92 ± 3.8	3.955	0.009*
40: 50 years	29	16.7	12.66 ± 3.9		
Over 50 years	126	72.4	10.19 ± 3.5		
**Gender**					
Male	94	54.0	10.48 ± 3.4	^t =^ 0.197	0.658
Female	80	46.0	10.73 ± 3.8		
**Marital state**					
Single	26	14.9	10.58 ± 3.0		
Married	138	79.3	10.63 ± 3.7	1.199	0.312
Divorced	7	4.0	8.71 ± 2.9		
Widowed	3	1.7	13.33 ± 0.57		
**Children number**					
0	38	21.8	10.79 ± 2.9		
1-3	48	27.6	11.0 ± 4.3	0.635	0.594
4-6	69	39.7	10.13 ± 3.2		
More than 6	19	10.9	10.84 ± 4.3		
**Are you responsible for your-self and family**					
Yes	138	79.3	10.56 ± 3.8	^t =^ 0.058	0.810
No	36	20.7	10.72 ± 2.5		
**Are you currently employed**					
Yes	29	16.7	9.90 ± 3.1	^t =^ 1.273	0.261
No	145	83.3	10.73 ± 3.7		
**Are you the family breadwinner**					
Yes	117	67.2	10.37 ± 3.5	^t =^ 1.362	0.245
No	57	32.8	11.05 ± 3.8		
**Is income adequate**					
Yes	157	90.2	10.46 ± 3.3	^t =^ 2.174	0.142
No	17	9.8	11.82 ± 5.4		
**Educational level**					
Secondary education or less	139	79.9	10.54 ± 3.4	^t =^ 0.143	0.706
University education or higher	35	20.1	10.80 ± 4.3		
**Do you undertake home activities**					
Yes	95	54.6	10.72 ± 3.9	^t =^ 0.241	0.624
No	79	45.4	10.44 ± 3.2		
**Residence location impact on sleep**					
Yes	40	23.0	9.88 ± 2.4	^t =^ 2.029	0.156
No	134	77.0	10.81 ± 3.9		
**Weight in KG** (mean±SD)	76.51 ± 17.07			1.850	0.002*

* Statistically significant at (p ≤ 0.05). F = ANOVA test. t-test, independent t-test.

Table 2 shows that less than half of the participants (41.4%) have a 2–3 litre fluid retention between sessions. Most of them (95.4%) have 3 sessions of dialysis each week. Over half of the participants spend 3.5 hours per session (56.9%). 41.4% of patients have the dialysis session during the morning. 68.4% of patients use a catheter for dialysis, and 67.2% of them didn’t have problems with the shunt. Body pain and hypotension were the most common conditions after the hemodialysis session (24.7%). 62.1% of the patients didn’t practice indoor or outdoor activities. Around one quarter of the patients (23%) smoke. 55.2% of patients perceived themselves to have average health status. 50.6% of patients have hypertension, and 73.6% suffered from pain. Only one third (33.3%) of patients take medications without a prescription. But more than half of the patients (55.2%) take a painkiller or other medication for sleep. Quality of sleep has a statistically significant relationship with the amount of fluid retention between sessions, problems/symptoms after session, perception of health status, intake of painkillers or medications for sleep (p = 0.041, 0.012, 0.001, 0.016), respectively.

**Table 2:** Health status Characteristics and their relation to sleep quality among the studied group (n = 174).

Variable	N	%	Sleep quality	F	P value
**Fluid retention between sessions**					
1-2 litres	32	18.4	9.44 ± 3.3	3.262	0.041*
2-3 litres	72	41.4	11.32 ± 3.3		
3-4 litres	70	40.2	10.37 ± 3.8		
**Hemodialysis Sessions per week**					0.504
2	8	4.6	9.75 ± 2.7	^t =^ 0.448	
3	166	95.4	10.63 ± 3.6		
**Number of hours per hemodialysis session**					
3 hours	43	24.7	11.21 ± 4.2		
3.5 hours	99	56.9	10.42 ± 3.4	0.839	0.434
4 hours	32	18.4	10.28 ± 3.3		
**Time of hemodialysis session during the day**					
Morning	72	41.4	11.18 ± 4.1	1.634	0.198
Afternoon	52	29.9	10.12 ± 3.3		
Evening	50	28.7	10.24 ± 3.1		
**Type of vascular access**					
Catheter	119	68.4	10.71 ± 3.8		
Natural	51	29.3	10.10 ± 3.1	1.616	0.202
Artificial	4	2.3	13.25 ± 4.5		
**Problems with shunt**					
Yes	57	32.8	10.74 ± 3.1	^t =^ 0.134	0.715
No	117	67.2	10.52 ± 3.9		
**Problems/symptoms after hemodialysis session**					
No	48	27.6	9.60 ± 2.8		
Body pain	43	24.7	9.79 ± 3.1		
Leg pain	8	4.6	11.63 ± 4.2	2.823	0.012*
Fatigue	28	16.1	10.54 ± 2.9		
Itching	1	0.6	10.0		
Hypotension & dizziness	43	24.7	12.30 ± 4.6		
Vomiting	3	1.7	11.33 ± 2.5		
**Activities indoor or outdoor**					
Yes	66	37.9	10.62 ± 3.9	^t =^ 0.007	0.934
No	108	62.1	10.57 ± 3.4		
**Smoking status**					
Yes	40	23.0	10.28 ± 3.2	^t =^ 0.393	0.532
No	134	77.0	10.69 ± 3.7		
**Perception of health status**					
Poor	56	32.2	12.02 ± 4.5	6.968	0.001*
Average	96	55.2	10.01 ± 2.9		
Good	22	12.6	9.50 ± 2.7		
**Chronic disease**					
No	23	13.2	9.74 ± 3.1		0.457
Hypertension	88	50.6	10.78 ± 3.9	0.914	
Diabetes	47	27.0	10.30 ± 3.2		
Cardio	10	5.7	11.10 ± 3.7		
Colon	6	3.4	12.5 ± 3.7		
**Currently suffering from pain**					
Yes	128	73.6	10.80 ± 3.6	^t =^ 1.541	0.216
No	46	26.4	10.02 ± 3.4		
**Pain assessment**					
Mild	52	29.9	9.77 ± 2.9	2.498	0.085
Moderate	52	29.9	10.54 ± 3.7		
Severe	70	40.2	11.24 ± 3.9		
**Over-the-counter Medications**					
Yes	58	33.3	10.33 ± 3.1	^t =^ 0.458	0.500
No	116	66.7	10.72 ± 3.8		
**Painkiller or medications for sleep**					
Yes	96	55.2	11.19 ± 3.9	^t =^ 5.901	0.016*
No	78	44.8	9.86 ± 3.1		

* Statistically significant at (p ≤ 0.05). F = ANOVA test. t-test, independent t-test.

The distribution of patients according to sleep quality items is presented in Table 3. 60.9% of patients go to bed early at night, 42% take 15–30 minutes to fall asleep each night. Patients usually wake up in the early morning (44.8%). Less than half of the studied population (47.1%) reported that they have 6–7 hours of sleep during the night. 36.2% of the subjects wake up in the middle of the night or early morning once or twice per week. 29.3% and 21.3% of the participants reported no symptoms of breathing difficulty and cough or snoring loudly, respectively, during the past month. Most of the patients on hemodialysis have reported disturbances in their quality of sleep in the form of having bad dreams over the past month (65.5%) and having pain (73.5%). 88.5% of patients had trouble staying awake while driving, eating meals, or engaging in social activity. Subsequently, only 9.8% rated the overall quality of their sleep as very good. Further, 85.6% have stated suffering from episodes of disorientation or confusion during the past month.

**Table 3:** Assessment of sleep quality among hemodialysis patients (n = 174).

1. During the past month, what time have you usually gone to bed at night?	No identified time	Early night		Late night
	29(16.7)	106(60.9)		39(22.4)
2. During the past month, how long (in minutes) has it usually taken you to fall asleep each night?	Less than 15 minutes	15-30 minutes	31-60 minutes	More than 60 minutes
	59(33.9)	73(42.0)	26(14.9)	16(9.2)
3. During the past month, what time have you usually gotten up in the morning?	Early morning (5–8am)	Late morning (9–12am)		Afternoon (1–4 pm)
	78(44.8)	19(10.9)		77(44.3)
4. During the past month, how many hours of actual sleep did you get at night? (This may be different than the number of hours you spent in bed).	More than 7 hours	6-7 h	5-6 h	Less than 5 hours
	8(4.6)	82(47.1)	43(24.7)	41(23.6)
5. During the past month, how often have you had trouble sleeping because you… a. Cannot get to sleep within 30 minutes	Not during the past month	Less than once a week	Once or twice a week	Three or more times a week
	15(8.6)	35(20.1)	79(45.4)	45(25.9)
b. Wake up in the middle of the night or early morning	20(11.5)	46(26.4)	63(36.2)	45(25.9)
c. Have to get up to use the bathroom	42(24.1)	60(34.5)	50(28.7)	22(12.6)
d. Cannot breathe comfortably	51(29.3)	57(32.8)	50(28.7)	16(9.2)
e. Cough or snore loudly	37(21.3)	59(33.9)	56(32.2)	22(12.6)
f. Feel too cold	63(36.2)	60(34.5)	37(21.3)	14(8.0)
g. Feel too hot	74(42.5)	61(35.1)	35(20.1)	4(2.3)
h. Have bad dreams	60(34.5)	67(38.5)	39(22.4)	8(4.6)
i. Have pain	46(26.4)	61(35.1)	44(25.3)	23(13.2)
j. Other reason(s)	71(40.8)	39(22.4)	47(27.0)	17(9.8)
6. During the past month, how often have you taken medicine to help you sleep (prescribed or “over the counter”)?	45(25.9)	69(39.7)	45(25.9)	15(8.6)
7. During the past month, how often have you had trouble staying awake while driving, eating meals, or engaging in social activity?	20(11.5)	37(21.3)	79(45.4)	38(21.8)
8. During the past month, how much of a problem has it been for you to keep up enough enthusiasm to get things done?	No problem at all	Only a very slight problem	Somewhat of a problem	A very big problem
	21(12.1)	72(41.4)	49(28.2)	32(18.4)
9. During the past month, how would you rate your sleep quality overall?	Very good	Fairly good	Fairly bad	Very bad
	17(9.8)	53(30.5)	88(50.6)	16(9.2)
10. Do you have a bed partner or roommate?	No bed partner or room mate	Partner/roommate in other room	Partner in same room but not same bed	Partner in same bed
	64(36.8)	34(19.5)	29(16.7)	47(27.0)
11. If you have a roommate or bed partner, ask him/her how often in the past month you have had: a. Loud snoring	Not during the past month	Less than once a week	Once or twice a week	Three or more times a week
	26(14.9)	60(34.5)	74(42.5)	14(8.0)
b. Long pauses between breaths while asleep	29(16.7)	52(29.9)	67(38.5)	26(14.9)
c. Legs twitching or jerking while you sleep	29(16.7)	50(28.7)	66(37.9)	29(16.7)
d. Episodes of disorientation or confusion during sleep	25(14.4)	60(34.5)	77(44.3)	12(6.9)

Table 4 presents the mean scores of the dimensions for sleep quality. Sleep efficiency dimension obtained the lowest mean score (0.89 ± 0.99) while sleep disturbance had the highest mean score. (10.74 ± 3.94). These findings reflect the very poor sleep quality among patients. It is worth noting that most of the patients on hemodialysis have a low quality of their sleep (91%).

**Table 4:** Mean scores of sleep quality dimensions among hemodialysis patients (n = 174).

Sleep quality dimensions	Mean	SD
Subjective sleep quality	1.59	0.79
Sleep latency	2.88	1.55
Sleep duration	1.67	0.88
Sleep efficiency	0.89	0.99
Sleep disturbance	10.74	3.94
Use of sleep medication	1.17	0.91
Day-time dysfunction	3.30	1.37
**Total Sleep quality dimensions**	10.59	3.63

Table 5 presents the correlation between blood biochemical values for patients on hemodialysis and the quality of their sleep. Low hemoglobin, hematocrit, sodium and ferritin levels were all statistically significantly correlated with poor quality of sleep among these patients (β = 0.794, 0.785, 0.240, 0.169), respectively.

**Table 5:** Correlation between biochemical values for patients and quality of their sleep (n = 174).

Variable	Mean	SD	β	t	P value
(Constant)				4.977	.000
Wbc	6.7678	2.91203	.026	.326	.745
Rbc	4.1814	.76118	.029	.271	.786
Hb	10.9256	1.60414	−.794	−3.149	.002*
Hematocrit	35.019	4.8424	−.785	−3.001	.003*
Platelets	230.580	86.7328	.061	.743	.458
Transferrin	1.7052	.34668	.068	.749	.455
Transferrin saturation	25.758	11.3740	.060	.715	.476
Urea	21.1406	6.60513	.107	1.013	.313
Creatinine	801.205	266.5781	−.051	−.538	.591
Albumin	39.397	3.6602	−.080	−.964	.336
Uric acid	392.632	98.0016	−.056	−.599	.550
Sodium	137.236	3.3585	−.240	−2.874	.005*
Potassium	5.288	.8557	−.074	−.863	.390
Bicarbonate	22.8034	3.72284	−.143	−1.560	.121
Phosphorus	1.8692	.64896	.124	1.404	.162
Magnesium	.8701	.13431	−.023	−.277	.782
Ferritin	526.40	375.627	−.169	−2.034	.044*

*P value is statistically significant

Table 6 demonstrates the correlation between the clinical characteristics of patients with the quality of their sleep. There is a positive correlation between sleep quality and problems/symptoms after hemodialysis session, as well as the presence of pain among studied patients (r = 0.282 & r = 0.168), respectively. In contrast, sleep quality has a negative correlation with patients’ perception of health status and taking painkillers or medications for sleep (r = −0.257, r = −0.182), respectively.

**Table 6:** Correlation between the clinical characteristics of patients and their sleep quality (n = 174).

Variable	Sleep quality	
Time of hemodialysis session during the day	r = −0.115	p = 0.131
Type of vascular access	r = −0.012	p = 0.875
Problems with shunt	r = −0.028	p = 0.715
Problems/symptoms after hemodialysis session	r = 0.282	p=0.001*
Smoking status	r = 0.048	p = 0.532
Perception of health status	r = −0.257	p=0.001*
Chronic disease	r = 0.088	p = 0.247
Currently suffering from pain	r = 0.168	p=0.026*
Over counter Medications	r = 0.052	p = 0.500
Painkiller or medications for sleep	r = −0.182	p=0.016*

* Statistically significant correlated at p ≤ 0.05 r=spearman correlation test.

In order to identify the independent characteristics of patients on hemodialysis that are related to poor quality of sleep, multiple linear regression analyses were conducted (Table 7). It was found that timing of the dialysis session per day and patients’ perception of health status were statistically significant independent negative predictors for poor sleep quality (β = 0.195, 0.337), respectively. In addition, problems/symptoms after hemodialysis sessions were statistically significant independent positive predictors of poor sleep quality (β = 0.212).

**Table 7:** Linear regression prediction model for sleep quality among studied patients.

Variables	B	Std. Error	Beta	t	P value	Lower Bound	Upper Bound
**(Constant)**	14.834	6.385		2.323	.021	2.221	27.448
**Working**	1.094	.790	.112	1.386	.168	−.466	2.654
**Enough income**	.755	.890	.062	.848	.398	−1.003	2.513
**Educational level**	.206	.688	.023	.300	.765	−1.153	1.565
**Weight**	−.005	.016	−.022	−.282	.779	−.037	.028
**Increasing session**	.356	.372	.072	.956	.340	−.379	1.091
**Sessions per week**	−.329	1.261	−.019	−.260	.795	−2.820	2.163
**Hours per session**	−.035	.853	−.003	−.041	.968	−1.719	1.650
**When you do dialysis per day**	−.853	.331	−.195	−2.576	.011*	−1.508	−.199
**Type of catheter**	−.028	.525	−.004	−.053	.958	−1.065	1.010
**Problems with shunt**	−.269	.617	−.035	−.436	.663	−1.488	.950
**Problems after session**	.384	.141	.212	2.724	.007*	.106	.663
**Smoking**	.494	.668	.057	.738	.461	−.827	1.814
**Health status**	−.604	.144	−.337	−4.204	.000*	−.888	−.320
**Chronic disease**	.232	.289	.058	.803	.423	−.339	.804
**Suffer from pain**	1.458	.913	.177	1.596	.112	−.346	3.262
**Medications without prescription**	.385	.586	.050	.657	.512	−.773	1.542
**Painkiller or medications for sleep**	−.311	.605	−.043	−.514	.608	−1.507	.885

R= 0.546 R^2^= 0.298 Adjusted R^2^ =0.212 F=3.449 ^*^Significance (p)=0.001

## Discussion

This study examined the magnitude and determinants of disrupted sleep patterns among patients on hemodialysis in the Kingdom of Bahrain. One of the major findings of this research project is the exceptionally high level of sleep disturbances in this population, with 91% reporting poor sleep quality, indicating a significant clinical and public health concern. Further, a detailed analysis of patients’ hemodialysis sleep habits revealed that most participants are suffering from sleep disruptions. Around half of the patients had six to seven hours of sleep per night, and only one in ten of these patients reported acceptable sleep quality. Patients described the irregular pattern of sleep, including sleep latency, sleep insufficiency, and nighttime arousals. These results are in line with earlier research that linked sleep impairments in patients on hemodialysis with the physiological and psychosocial demands of CKD and hemodialysis. Poor sleep is acknowledged to be widespread among patients on hemodialysis and should be prioritized by healthcare professionals for early identification and management [27].

Among the sociodemographic variables examined in this study, age and weight of the patients were statistically significant predictors of sleep quality. Older age is reported to be associated with changes in circadian rhythms and an increase in comorbidities, both of which are contributors to altered sleep patterns. Despite the majority of participants being unemployed and more than half being male, employment status and gender were not independently linked to sleep quality. Over half of the patients used medications or painkillers to aid sleep, which reflects unrecognized psychological distress among these patients. This emphasizes the need for routine mental health assessments in dialysis settings, a finding supported by previous studies [28–29], which demonstrated that frailty, age, and dialysis duration significantly influenced sleep outcomes. This highlights the importance of mental health integration into nephrology care in Bahrain. Literature suggests the importance of family support and marital status in sleep quality among patients on hemodialysis (9). However, in this study, marriage does not appear to be related to the quality of sleep in patients on hemodialysis. A possible explanation for this discrepancy could be that in this community, a working spouse may not always be available for emotional or logistical support since many families rely on domestic workers and nurses to provide caregiving for patients with chronic illnesses in general, and those on hemodialysis in particular.

Dialysis-related factors also played a role in sleep disturbances. Significant associations were observed between sleep quality and interdialytic weight gain, post-dialysis complications, and dialysis timing. Although morning sessions were the most common, sleep quality did not differ significantly across session times. However, body pain and hypotension were the most frequently reported post-session symptoms, both of which are known contributors to sleep disruption. These findings are consistent with global studies [28–29] highlighting the impact of dialysis-related symptoms on sleep. Pruritus is a frequent comorbidity in dialysis patients, significantly impairs sleep and often coexists with other complications, including electrolyte imbalances and cardiovascular instability [30]. Similarly, Öz et al. recently emphasized that pain severity, which is often influenced by diabetes, low education levels, and elevated inflammatory markers, substantially contributes to increased symptom burden and reduced sleep quality among patients on hemodialysis [31].

Clinical and biochemical predictors of poor sleep were also evaluated in this study. Multivariate regression analysis identified hemoglobin, hematocrit, sodium, and ferritin levels as significant factors influencing sleep. Higher hemoglobin and hematocrit levels were particularly associated with better sleep outcomes, reinforcing the role of anemia management in improving sleep quality among patients on hemodialysis. Furthermore, the role of self-perceived well-being in sleep health was demonstrated through the results, which revealed that self-rated health status was inversely related to sleep disturbance. This finding is aligned with published literature that shows that patients with anemia, pruritus, hypertension, decreased estimated glomerular filtration rate (eGFR) levels, aged over 50 years, and those with a longer disease duration are more likely to have worse sleep outcomes [32–33]. Moreover, the duration of dialysis, creatinine, and urea levels were not associated with sleep quality, suggesting that the effectiveness of hemodialysis may not be an independent factor in sleep disorders among patients on hemodialysis in this population. Future research is needed to clarify the association between hemodialysis effectiveness and sleep quality.

Physical functioning is another contributor to sleep quality. Research by Abforoushha et al. found that older patients on hemodialysis with low muscle strength were more prone to poor sleep [34]. This creates a cycle of physical decline and sleep disturbance, as sleep deprivation can also impair muscle recovery. This relationship calls for urgent attention, as most patients on hemodialysis in Bahrain are over 50 years. Sułkowski et al. further emphasized the impact of fatigue, which is a common symptom of anemia, on overall quality of life in such patients [35]. Moreover, over half of the patients rated their sleep as poor, and around four out of ten patients reported daytime drowsiness. This is despite having most patients living in environments that did not disturb sleep and had limited exposure to smoking or room temperature fluctuations. These findings suggest that even minimal sleep disturbances can have impactful consequences. Interventional programs that focus on behavioral changes, such as sleep hygiene education and relaxation techniques, like breathing exercises, may be beneficial in this situation. Almutary and AlShammari have demonstrated that non-pharmacological interventions can improve both mental health and sleep quality in patients on hemodialysis [36]

Collectively, these results stress the importance and need for a multidisciplinary approach in Bahrain’s nephrology clinics. Several interventions should be implemented, including nutritional support, routine biochemical monitoring, anemia management, education on sleep hygiene, and physical rehabilitation. Integrating these services into hemodialysis units would reflect a proactive model of care that aligns with international best practices.

It’s worth noting that the use of regression modelling in the analysis revealed that self-perceived poor health and the timing of dialysis were independently significantly associated with worse sleep quality. It would appear that improving patient outcomes heavily relies on ensuring dialysis adequacy. This is supported by the findings of Esmaeili et al. [37], who emphasized that in order to achieve reduced symptom burden, better sleep quality, and improved biochemical profiles, therefore, adequate dialysis delivery should be implemented. While there are few published reports in the Arab Gulf Region on sleep disturbances in patients on dialysis, this study in Bahrain is the first to be conducted in this population. One of the main strengths of this study in the Kingdom of Bahrain is that it employed univariate as well as multivariate analysis, which certainly contributes to the understanding of this topic through addressing a significant knowledge gap in regional nephrology research and contributing valuable data that supports global understanding of the sleep challenges faced by patients on hemodialysis. Another strength of this research is the application of a comprehensive approach throughout the study, which incorporates sociodemographic, clinical, behavioral, and biochemical variables. This framework allows for a more detailed analysis of the factors influencing sleep quality and helps to identify both modifiable and non-modifiable risk factors. Further, the use of multiple linear regression modelling and correlation analysis strengthens the validity of the findings. Independent predictors of poor sleep quality were identified through the use of these methods, which supports more precise and evidence-based clinical recommendations. Finally, this study identifies multiple factors that can be assessed and managed in clinical practice to improve sleep quality. This includes the association between sleep quality and anemia, post-dialysis symptoms, and perceived health status. These findings allow the exploration of different techniques to improve dialysis care. This includes using targeted strategies such as patient monitoring, managing anemia, and psychosocial support.

Although this study offers valuable insights, it has some drawbacks. A cross-sectional design was used, which limits the ability to draw conclusions about causality. In addition, the data were obtained based on self-reports from patients, which may introduce reporting bias, particularly for subjective variables such as sleep quality and health perception. We propose using objective measures of sleep, such as polysomnography, in future research to enhance accuracy.

We recommend that future research should incorporate longitudinal studies to explore causality and track changes over time. In addition, it would be beneficial to test interventions that focus on sleep improvement strategies for patients on hemodialysis. Further, comparative studies between the Arab Gulf area and other regions may reveal the influence of cultural and healthcare system differences on sleep outcomes.

## Conclusion

The study reveals that most patients on hemodialysis (91%) are suffering from poor sleep quality. The key determinants are multifactorial, including age, weight, anemia, post-dialysis symptoms, and perceived health. Behavioral and psychosocial indicators, such as medication use, fatigue, and self-rated health, were significantly associated with poor sleep quality. In this context, these findings highlight the importance of integrating sleep assessments, mental health screening, and patient education programs into routine nephrology care. Furthermore, ensuring dialysis adequacy remains essential, not only for disease management but also for improving sleep and overall quality of life.
